# Supplementary material for: HIVprotI: an integrated web based platform for prediction and design of HIV proteins inhibitors
Source: J Cheminform. 2018 Mar 9;10:12. doi: 10.1186/s13321-018-0266-y (PMC5845081; doi:10.1186/s13321-018-0266-y)
Supplement: Supplementary file 2 — Additional file 2. Source code of HIVProtI web server. [file 13321_2018_266_MOESM2_ESM.zip › HIVprotI_Source-code/hivprotI/weka-3-6-6/doc/weka/core/Copyright.html]

Copyright


---


|  |  |  |
| --- | --- | --- |
| **Overview** \| **Package** \| **Class** \| **Tree** \| **Deprecated** \| **Index** \| **Help** \| **Weka's home** | |  |
| **PREV CLASS**   **NEXT CLASS** | **FRAMES**    **NO FRAMES**     **All Classes** |  |
| SUMMARY: NESTED \| FIELD \| CONSTR \| METHOD | DETAIL: FIELD \| CONSTR \| METHOD |  |


---


## weka.core Class Copyright

```
java.lang.Object
  weka.core.Copyright
```

---

``` public class Copyright extends java.lang.Object ```

A class for providing centralized Copyright information.

**Version:**
:   $Revision: 1.2 $

**Author:**
:   fracpete (fracpete at waikato dot ac dot nz)

---

| **Field Summary** | |
| --- | --- |
| `static java.lang.String` | `PROPERTY_FILE`             the copyright file |


**Constructor Summary**

`Copyright()`


| **Method Summary** | |
| --- | --- |
| `static java.lang.String` | `getAddress()`             returns the address of the owner |
| `static java.lang.String` | `getFromYear()`             returns the start year of the copyright |
| `static java.lang.String` | `getOwner()`             returns the entity owning the copyright |
| `static java.lang.String` | `getToYear()`             returns the end year of the copyright (i.e., current year) |
| `static java.lang.String` | `getURL()`             returns the URL of the owner |
| `static void` | `main(java.lang.String[] args)`             Only for testing |

**Methods inherited from class java.lang.Object**

`equals, getClass, hashCode, notify, notifyAll, toString, wait, wait, wait`

**Field Detail**

### PROPERTY\_FILE

```
public static final java.lang.String PROPERTY_FILE
```

:   the copyright file

    **See Also:**: Constant Field Values


**Constructor Detail**

### Copyright

```
public Copyright()
```


**Method Detail**

### getFromYear

```
public static java.lang.String getFromYear()
```

:   returns the start year of the copyright

    :   **Returns:**: the start year

---


### getToYear

```
public static java.lang.String getToYear()
```

:   returns the end year of the copyright (i.e., current year)

    :   **Returns:**: the end/current year

---


### getOwner

```
public static java.lang.String getOwner()
```

:   returns the entity owning the copyright

    :   **Returns:**: the owner

---


### getAddress

```
public static java.lang.String getAddress()
```

:   returns the address of the owner

    :   **Returns:**: the address

---


### getURL

```
public static java.lang.String getURL()
```

:   returns the URL of the owner

    :   **Returns:**: the URL

---


### main

```
public static void main(java.lang.String[] args)
```

:   Only for testing

    :   **Parameters:**: `args` - ignored


---


|  |  |  |
| --- | --- | --- |
| **Overview** \| **Package** \| **Class** \| **Tree** \| **Deprecated** \| **Index** \| **Help** \| **Weka's home** | |  |
| **PREV CLASS**   **NEXT CLASS** | **FRAMES**    **NO FRAMES**     **All Classes** |  |
| SUMMARY: NESTED \| FIELD \| CONSTR \| METHOD | DETAIL: FIELD \| CONSTR \| METHOD |  |


---
